# Crowding in the City: Losing and Winning Competitors of an Invasive Bird

**DOI:** 10.1371/journal.pone.0100593

**Published:** 2014-06-19

**Authors:** Dailos Hernández-Brito, Martina Carrete, Ana G. Popa-Lisseanu, Carlos Ibáñez, José L. Tella

**Affiliations:** 1 Department of Conservation Biology, Estación Biológica de Doñana, Consejo Superior de Investigaciones Científicas (CSIC), Sevilla, Spain; 2 Department of Physical, Chemical and Natural Systems, University Pablo de Olavide, Seville, Spain; 3 Leibniz Institute for Zoo and Wildlife Research, Berlin, Germany; 4 Department of Evolutionary Ecology, Estación Biológica de Doñana, Consejo Superior de Investigaciones Científicas (CSIC), Sevilla, Spain; Phillip Island Nature Parks, Australia

## Abstract

Invasive species can take advantage of resources unexploited by natives (opportunism hypothesis) or they can exploit the same resources but more aggressively or efficiently (competition hypothesis), thus impacting native species. However, invasive species tend to exploit anthropogenic habitats that are inefficiently used by natives such as urban environments. Focusing on the ring-necked parakeet (*Psittacula krameri*), one of the most invasive birds worldwide, we combined observations of interspecific aggressions, species-specific cavity-nest preferences and the spatial distribution of the native cavity-nesting vertebrate community to determine the invasion process as well as its potential impacts on native species in a Mediterranean city. Our results support the competition hypothesis, suggesting that ring-necked parakeets are outcompeting native species sharing nest-site preferences. Parakeets initiated and won most interspecific aggressions, which were directed towards competitors but also towards predators. This behaviour could explain the spatial arrangement of natives, with most bird species breeding close to parakeets possibly to take advantage of their effective antipredatory behaviour. However, temporal and spatial patterns of segregation suggest that a threatened bat species is negatively affected by parakeets. This demonstrates that common species gain benefits and threatened ones (in this study, a bat and possibly a falcon) lose nest sites due to invaders. Therefore, the conservation status of the native species that pay the costs of competition with invaders should be considered. This scenario of winners and losers may, however, shift towards more losers if the ring-necked parakeet population continues to grow, thus requiring close monitoring and control/eradication programs to avoid further impacts.

## Introduction

Biological invasions are considered a major threat to global biodiversity, since invasive species may cause negative impacts on natives through increased predation risk, competition, hybridization or the spread of disease [1]. At smaller scales, however, the relationship between invasive species and biodiversity measures is less clear [2], as introduced species can contribute to species gain by its establishment [3], can reduce species richness through extinction processes [4] or can have no detectable effects on native biota [5]. These different patterns may be explained by the nature of the invader [6] but also by the characteristics of the recipient community [7]. In an opportunistic scenario (formally called the empty niche, the invasion window or the opportunity window hypotheses [7]), invasive species are functionally different from species already present in the community and thus their entrance into a new environment can occur without the displacement or extinction of natives. Conversely, when exotic and native species exploit similar resources, the recipient community could resist an invasion as a result of competition that stems from high local diversity and low niche vacancy [8], [9]. However, if exotic species are able to out-compete natives by exploiting resources more efficiently or through aggressive behaviours, they can successfully invade the new area causing the displacement of the native competitor [10].

Urban environments represent a challenge to biodiversity, as not all native species inhabiting the surrounding rural habitats are able to colonize these areas [11]. Different studies have found a reduction in richness and diversity of native species along urban gradients, often in parallel with increments in exotic invasive ones [12]. Thus, as cities expand across the globe, biological homogenization increases as a consequence of the widespread increment of urban-adaptable, often invasive species at the expense of native, often endemic ones [13]. This pattern suggests that many exotic and native species may not compete in nature [14], as the former tend to be particularly abundant in habitats that are inefficiently used by natives, such as in urban environments [15]. However, cities still serve as refuges and conservation areas for some endangered natives [16], which might come into conflict with invasive species using highly similar resources.

Ring-necked parakeets (*Psittacula krameri*) are native to Asia and Africa and have established non-native urban populations in at least 35 countries on five continents [17]. Although it is considered amongst the 100 worst alien species in Europe (http://www.europe-aliens.org/speciesTheWorst.do), its impact on native species remains unclear. The ring-necked parakeet requires medium-size (4–8 cm entrance size [18]) natural cavities or those excavated in trees by other species for breeding. Given the usual shortage of tree cavities [19], especially in urban environments where decaying tree limbs are periodically removed in the interests of public safety [20], parakeets could outcompete native cavity-nesting species in aggressive interactions and thus spread at the cost of the numbers and/or distribution of natives (competition hypothesis). Alternatively, if the native community is poor in secondary cavity nesters and/or the resource is not limited, the establishment of this invasive species could be facilitated by a high availability of nesting sites (opportunism hypothesis). Previous work has shown that parakeets can outcompete only one of the coexisting native cavity-nesting bird species in a central-European city [18], [21], but larger-scale studies comparing areas occupied and not occupied by this invader suggest little or no impact on populations of native birds [22], [23].

The outcome of invasive-native competition could be context-dependent, being influenced by the availability of resources and the composition of the native community [7]. Thus, answering similar questions but using different systems can help to make generalizations about processes from local patterns. Here, we combined observations of interspecific aggressive interactions, species-specific cavity-nest preferences and the spatial distribution of cavities available and used by each species to infer the process as well as the consequences of ring-necked parakeet invasions in a Mediterranean city. Results show a complex scenario where, although ring-necked parakeets outcompete native species in aggressive encounters, most natives seem to benefit from the effective anti-predator behaviour of parakeets. Conversely, some threatened native species can be displaced by parakeets, resulting in a dynamic process of winners and losers linked to the population growth of the invader.

## Methods

### Ethics Statement

Field work conducted here was not invasive and did not require the manipulation of live animals. Therefore, this work did not require specific permits by the relevant Spanish authorities.

### Study System

The ring-necked parakeet was a commonly traded wild species for the Spanish cage-bird market [24] and a number of urban populations arose largely from accidental escapes from cages [25]. This study focuses on the city of Seville (southern Spain), where the first records of the species date back to the early 1990’s and the initially small population sharply increased [Authors’ unpublished data], reaching ca. 1,000 individuals in 2011 (P. Edelaar com. pers.). We conducted the first breeding census of the species in the city of Seville from March to July 2013. We first located potential breeding areas where the species was present taking advantage of its conspicuous behavior. Then, we monitored the available cavities to assess whether or not they were occupied by parakeets based on the observation of adults entering a minimum of 10 times on different days, the vocalizations of chicks inside the nest, and/or the observation of juveniles at the entrance. We located 216 active nests, 159 (73.6%) in an urban park (the María Luisa Park; 37° 22′ 31.57" N, 5° 59′ 19.59" W) and the rest forming smaller breeding nuclei in scattered groups of trees or, more rarely, in buildings throughout the city. María Luisa is the largest park located in the core of the city, comprising a 40 ha wooded area with a variety of tree species, most of them exotics such as *Platanus* sp., *Eucalyptus* sp. or *Gleditsia triacanthos*. The park is completely surrounded by streets with moderate to high traffic intensity.

### Availability and Occupancy of Tree Cavities

The assessment of the availability of tree cavities and their occupancy by parakeets and native species was restricted to María Luisa Park to avoid potential biases when analysing interspecific competition (e.g., small groups of trees outside of the park occupied by parakeets could not be occupied by some native species because they did not offer sufficient foraging habitat). We GPS located (±3 m) all tree cavities that we were able to visually inspect in trees located within the park by using 10×50 binoculars. In each case, we identified the tree species and estimated the height of the cavity above ground (in m) and the width of its entrance (in cm). The entrances of cavities were categorized as small, medium or large (<4 cm, 4–8 cm, and >8 cm, respectively) according to previous studies, which showed the preference of parakeets for cavities with entrances between 4 and 8 cm width [21], [26]. Cavities located at <2.5 m above ground were not considered for analyses since their accessibility to humans would preclude its use (none were occupied by native or exotic species), thus biasing results.

From January to August (covering the entire breeding season of native and exotic species), we repeatedly visited and observed at a distance (for a minimum of 10 min) each cavity on at least 10 different days during daylight hours to assess whether or not it was occupied and by what species, devoting 48 days (202.5 hours) of field work. The close proximity of many trees with cavities often allowed us to monitor several trees simultaneously. A cavity was considered as occupied by a given bird species when we observed adults entering a minimum of 10 times on different days, heard chicks inside, or observed juveniles at the entrance. In addition, María Luisa Park is also inhabited by the greater noctule (*Nyctalus lasiopterus*). This cavity-breeding forest species is the largest European bat (averaging 48 g [46]), and the whole population living in and around Seville (roughly estimated at ca. 500 individuals in 2003–2004) gathers to breed and roost communally in the tree cavities of this park [27], [28]. To identify the cavities used by greater noctules, we detected their presence using an ultrasound detector (Pettersson D 230) and observing bats leaving tree cavities at sunset. Greater noctules, like other forest bats, form fission-fusion societies that switch roosts every few days, so each bat colony can control a large number of roosts of which only a few are occupied at a specific time [27]. Thus, using previous information on radio-tracked individuals [27], we considered that a tree was not used by greater noctules during spring-summer 2013 if we did not observe activity during any of our 10 spaced visits. Using information on trees used by noctules during 2003–2004, we also tested for changes in their use in relation to the current nesting spatial distribution of ring-necked parakeets. These trees were located after monitoring 27 noctules through radio-tracking to study the spatial pattern of tree use by the species, finding that cavities located in 75 trees were alternatively used as roost sites ten years ago [27]. It is worth noting that the different methodologies used to identify occupied trees could produce false cases of inoccupation in both 2003–2004 (a larger period of time monitoring 27 individuals) and 2013 (a shorter period of time monitoring all tree cavities). However, it may also just produce statistical noise making our estimates conservative.

### Spatial Distribution of Occupied Cavities

The occupancy of a particular tree cavity by a given species could be influenced by the spatial distribution of cavities occupied by the same and/or other species, driven not only by competition but also by conspecific and heterospecific attraction processes. We thus obtained the distance from each occupied cavity to the nearest cavity occupied by conspecifics and heterospecifics (nearest neighbour distance) as well as the corresponding nest aggregation indexes. These aggregation indexes were obtained as the relative position of each occupied cavity within the whole distribution of all cavities occupied by conspecifics or heterospecifics in the park using *∑ exp (-d_ij_)*, with (*i≠j*) where *d_ij_* is the linear distance between occupied cavities *i* and *j*, *j* representing all occupied cavities [29]. These variables were complementary measures depicting the social environment around each nest cavity at a landscape scale as well as the existence of close competitors.

The spatial distribution of occupied cavities could also be influenced by habitat heterogeneity in the park. We considered the two main sources of habitat heterogeneity in our study area, i.e. the proximity to surrounding streets and forest cover. Noise from car traffic could alter song performance, reproductive success and even the spatial distribution of birds [30], [31]. We therefore measured the linear distance from each cavity to the closest street using GIS tools (see below). On the other hand, species could differ in their preferences for forest coverage around cavities. We obtained forest cover by measuring it in a radius of 30 m around each GPS located cavity on a Google Map image taken in 2013 (Imagens ©2013 Cnes/Spot Image, DigitalGlobe, Instituto de Cartografía de Andalucía, map data ©2013 Google, based on BCN IGN Spain), using OpenLayers Plugin (1.1.0) applications in Q-GIS 1.8.0 (2008 Free Software Foundation, Inc). Forest cover was then scored into four main categories, namely 0–25%, 25–50%, 50–75%, and 75–100%.

### Interspecific Interactions

We assessed interspecific interactions by randomly sampling the behaviour of different nesting ring-necked parakeets during a 15-minute period. We conservatively recorded the bird species present within a radius of 15 m around the focal parakeet, whether or not there was an aggressive interaction, what species started the attack, and which was the winner. To increase sample size without resampling the same individuals or the number of potential interacting species, observations were conducted in María Luisa Park as well as in other urban areas of Seville occupied by the species (see above), totaling 88 days (351.5 hours) of field work. These areas included the main parks of the city as well as a church (Divino Salvador) where ring-necked parakeets occupied cavities in walls for breeding, potentially competing there with lesser kestrels (*Falco naumanni*), a colonial falcon that usually breeds in urban buildings [32].

### Statistical Analysis

We employed Generalized Linear Models (GLM) implemented through the GENMOD procedure in SAS 9.2 [33] to ascertain which variables determined hole occupation, using the binomial error distribution (cavity occupied or not occupied by a given species) and the logistic link function. In a first set of models, we aimed to determine whether occupied and vacant cavities differed in their structural characteristics. Thus, we modelled the probability of occupancy as a function of the height of the cavity above ground (in its linear and quadratic forms), the entrance size, and the tree species. The resulting species-specific patterns of cavity preferences (see results) made it difficult to identify similarities (and thus opportunities for competition) between species. Therefore, we performed a categorical principal component analysis (CATPCA) on entrance size (since it is a categorical variable) and height above ground of the cavities occupied and took the scores of the obtained first dimension as a single compiling descriptor of the cavities used by each species. An ANOVA on these scores allowed us to identify differences in cavity preferences among species, and post-hoc Scheffe tests permitted us to establish homogeneous groups (i.e., species not differing in their preferences for particular cavity traits). In a second set of models, we assessed the spatial arrangement of each species regarding cavity traits, the distribution of both conspecifics and heterospecifics, and main habitat features (distance to the nearest street and forest cover) around each occupied and available (i.e., unoccupied) cavity, also using GLMs with a binomial error distribution and logistic link function.

Exact binomial tests were used to assess whether the proportion of interspecific encounters ending in aggressions, the proportion of aggressions initiated by ring-necked parakeets, and the proportion of aggressions won by this species differed significantly from parity. To obtain interspecific patterns in the frequency of aggressions and their outputs, we also used GLMs with a binomial error distribution and a logistic link function, fitting as explanatory variables the average body mass of the species interacting with ring-necked parakeets (obtained from [34]), their overlap in nest-site preferences (as a factor with levels ranging from 0– the interacting species was not a cavity-nester- to 3– maximum overlap in nest-site traits), and whether the interacting species was a potential predator of eggs, nestlings or adults. We expected that ring-necked parakeets would be more prone to attack those species with overlapping nest-site preferences and potential predators, and less prone to attack larger-bodied species.

A backward procedure was performed for GLM modelling, removing from full models those variables that were non-significantly associated with the response variable (p>0.05) to obtain minimum adequate models (MAM) [15]. The resulting models did not show data overdispersion. We calculated the percentage of deviance explained as a measure of the variance explained by each MAM.

## Results

### Occupancy of Tree Cavities

We recorded 1,086 cavities in 435 trees located within María Luisa Park during the 2013 breeding season. Cavities were located at an average height above ground of 13.09 (SD 5.51) m, and the commonest cavity entrances (47%) were of intermediate size (4–8 cm). Most cavities (62.2%) were located in London plane trees (*Platanus* × *acerifolia*) probably because it is the most abundant species within the park but is also the species with highest number of available cavities.

A total of 10 species were found occupying 525 cavities (Table 1), including 9 bird and one bat species. Two bird species were exotics, i.e. the ring-necked parakeet and the blue-crowned parakeet (*Aratinga acuticaudata*). Ring-necked parakeets, feral pigeons (*Columba livia* var. *domestica*), house sparrows (*Passer domesticus*), and greater noctules showed the largest percentages of occupied cavities, while the rest of the species used less than 10% of occupied cavities (Table 1).

**Table 1:** Number and percentage of cavities occupied by each species during the 2013 breeding season in María Luisa Park (Seville, Spain).

Species	N of occupied cavities	%
BIRDS		
Ring-necked parakeet (*Psittacula krameri*)	159	30.29
Blue-crowned parakeet (*Aratinga acuticaudata*)	2	0.38
Tawny owl (*Strix aluco*)	1	0.19
Feral pigeon (*Columba livia* var. *dom.*)	133	25.33
Geat tit (*Parus major*)	13	2.48
Blue tit (*Cyanistes caeruleus*)	9	1.71
Short-toed treecreeper (*Certhia brachydactyla*)	2	0.38
House Sparrow (*Passer domesticus*)	105	20.00
Spotless starling (*Sturnus unicolor*)	45	8.57
BATS		
Greater noctule (*Nyctalus lasiopterus*)	56	10.67
TOTAL	525	

### Species Partitioning of Tree Cavities

**Figure 1:**
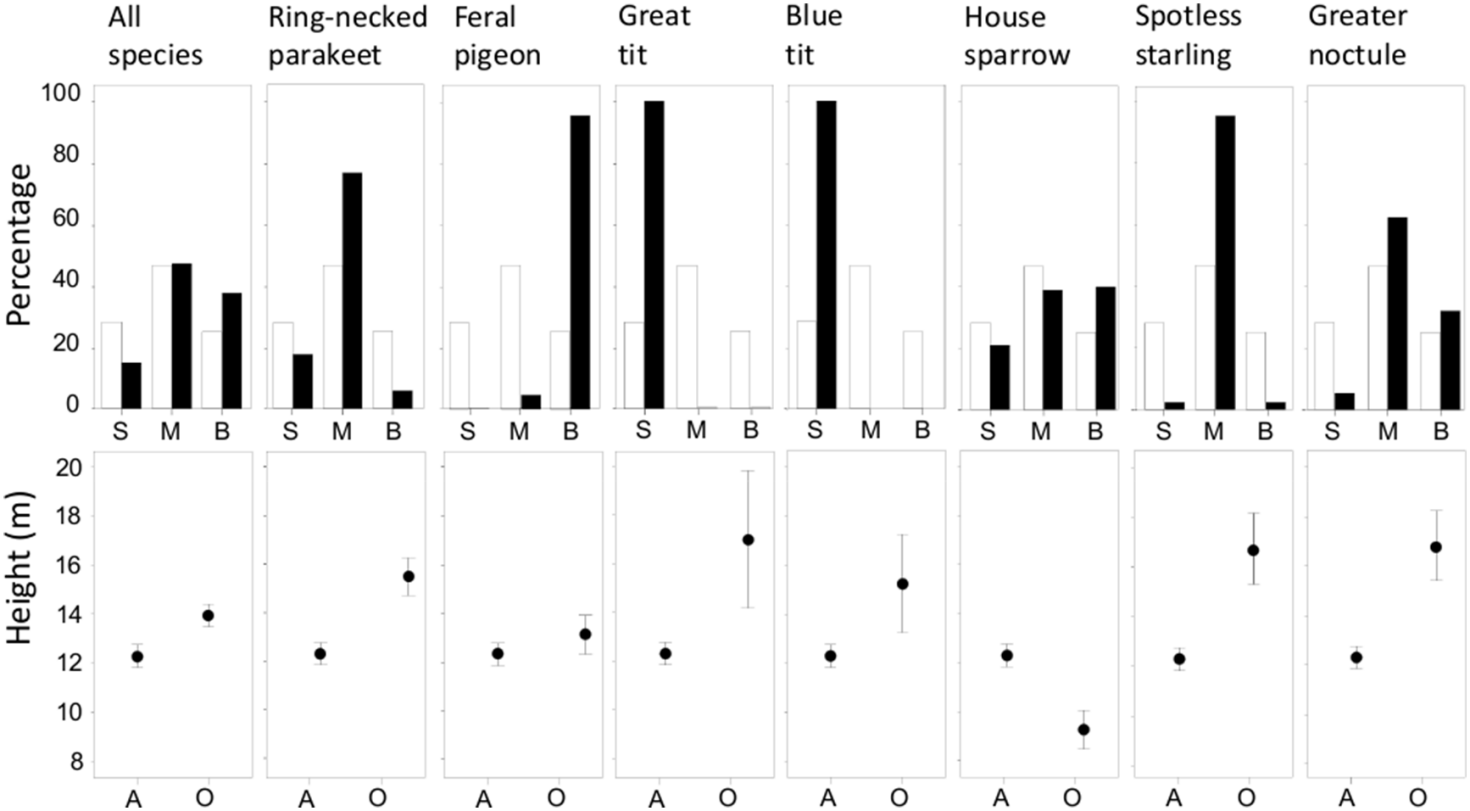
Differences in cavity size (small, medium, and big) and height above ground (mean and 95% CI) between tree holes occupied (black bars) and available (white bars) during the 2013 breeding season. All figures are depicted at the same scale to allow easier inter-specific comparisons.

More than half of the cavities (51.7%, *n* = 1,086) were unoccupied during the study period. However, occupied cavities significantly differed from unoccupied ones in terms of entrance size and height above ground, both considering all species together and each species from which the sample size allowed us to build separate GLMs (Table 2). Figure 1 illustrates the direction of the effects. Except for house sparrows, most species seemed to prefer cavities located at greater heights than those available (i.e., unoccupied by any species). Entrance size of occupied and available cavities also varied among species. Great (*Parus major*) and blue tits (*Cyanistes caeruleus*) only used small-size cavities, feral pigeons made more use than expected of the large ones, while ring-necked parakeets, spotless starlings (*Sturnus unicolor*) and greater noctules seemed to prefer medium-sized cavities. Although traits of occupied and available cavities also differed significantly in the case of house sparrows (Table 2), this species was distributed more evenly, nesting in cavities of different sizes (Figure 1). Tree species and the interactions among variables did not predict cavity occupancy by any species, since these terms were not retained in the MAM (Table 2).

**Table 2:** GLMs obtained to explain the probability of cavity occupancy by a given species and by all species together as a function of cavity traits (entrance size and height) and tree species.

	Size		Height		% dev
	χ^2^_ (df = 2)_	P	χ^2^_ (df = 1)_	P	
All species	37.38	<0.001	26.07	<0.001	4.08
Ring-necked parakeet	40.97	<0.001	25.50	<0.001	10.52
Feral pigeon	256.60	<0.001	9.11	0.0025	38.19
Great tit	32.85	<0.001	8.31	0.0039	32.62
Blue tit	22.45	<0.001			24.26
House sparrow	6.65	0.036	30.21	<0.001	6.82
Spotless starling	40.60	<0.001	15.23	<0.001	19.86
Greater noctule	16.65	0.0002	27.79	<0.001	12.18

The number of cavities occupied by each species is reported in Table 1, and the number of unoccupied (available) cavities was 561. % dev: percentage of deviance explained.

The first dimension obtained in a CATPCA (eigenvalue = 1.12) explained 56.1% of variance of the combined traits of occupied cavities, positively correlating with entrance size (*r* = 0.99) and negatively with height above ground (*r* = −0.21). Using the scores of this dimension as a single descriptor of cavity traits, we found significant differences among cavities occupied by the different species (ANOVA *F*_6,513_ = 69.99, *P*<0.001). A post-hoc Scheffe test identified three homogenous subgroups where the species belonging to each one did not differ in the characteristics of the cavities used: 1) ring-necked parakeet, spotless starling and greater noctule (*P* = 0.92), 2) great and blue tits (*P* = 0.99), and 3) feral pigeon and house sparrow (*P* = 0.93).

### Spatial Arrangement of Species

The above results suggest evidence for competition for certain kinds of cavities within the three subgroups of species considered. However, the actual occupancy of cavities by each species may also be influenced by the spatial distribution of conspecifics and heterospecifics, through social interactions that may range from agonistic encounters to hetero- and conspecific attraction, and by habitat features around cavities. Models considering the distance to the nearest occupied cavity (D) and the surrounding aggregation of occupied cavities (A) by conspecifics or heterospecifics, while controlling for habitat features (Table 3), were better to explain the probability of cavity occupancy (see % of deviance explained) than those just relying on cavity traits (Table 2). While habitat features were only related to the spatial distribution of three species (great tit, house sparrow, and spotless starling), all species seemed to be influenced by the spatial distribution of other birds, and in two species (blue tit and house sparrow) some cavity traits even dropped from models when the social environment was taken into account (Table 3). The probability of cavity occupancy decreased at greater distances from conspecifics (D_intra_) in all species except the blue tit, which tended to avoid large conspecific aggregations (A_intra_).

**Table 3:** GLMs explaining the occupancy of cavities by each species as a function of cavity traits, cover of forest canopy, distance to the nearest street, distance to the nearest occupied cavity (D), and aggregation of occupied cavities around the focal one (A).

	Cavity size	Cavity height	Forest cover	Distance street	Dintra	Aintra	Dinter	Ainter	% dev
**Ring-necked parakeet**	35.58***	4.75*			144.01 (−)***			7.89 (−)***	29.47
**Feral pigeon**	243.54***	18.27***			7.36 (−)***	65.79 (−)***	27.92 (−)***	42.81 (+)***	51.04
**Great tit**	15.85***	12.93***		5.27 (−)*	6.01 (−)*	35.31 (−)***		15.35 (+)***	68.11
**Blue tit**	16.87***					49.99 (−)***		27.25 (+)***	78.59
**House sparrow**		21.08***	13.51***	10.97 (+)***	31.04 (−)***		15.71 (−)***	21.04 (−)***	31.74
**Spotless starling**	37.41***	18.01***		15.14 (+)***	4.58 (−)*	62.82 (−)***	12.41 (−)***	4.48 (+)*	48.45
**Greater noctule**	17.02***	18.55***			40.14 (−)***		5.69 (+)**		22.91

Variables describing social environments (A and D) were obtained considering cavities occupied by the same species (intraspecific; A_intra_, D_intra_) and by the ring-necked parakeet (A_inter_, D_inter_). The species involved in interspecific significant effects (A_inter_, D_inter_) was always the ring-necked parakeet, except in the case of the model obtained for this species, where A_inter_ corresponds to the aggregation regarding greater noctules. Signs between brackets indicate positive or negative effects of variables on the probability of cavity occupancy. χ^2^ values are given for each variable; *: p<0.05, **: p<0.01, ***p<0.001; % dev: percentage of deviance explained.

Interspecific effects on spatial distributions differed among species (Table 3). For most bird species, the probability of cavity occupancy increased at closer distances to the nearest cavity occupied by ring-necked parakeets and/or the larger the aggregation of the invader (except for house sparrows, which seemed to avoid large aggregations of ring-necked parakeets). However, cavity occupancy by greater noctules was higher the greater the distance to cavities occupied by ring-necked parakeets, and cavity occupancy by ring-necked parakeets was lower the greater the spatial aggregation of greater noctules, thus suggesting a process of spatial segregation between these two species.

### Temporal Changes in the Spatial Distribution of Greater Noctules

As the available data for the period 2003–2004 was restricted to trees holding cavities occupied by greater noctules, our analyses of changes in occupancy were done at the tree scale. From 75 trees occupied 10 years ago, 49 were unoccupied by noctules in 2013 despite they still offered suitable cavities, which implies a loss of ca. 39% of occupied trees during the last 10 years. The probability that a tree was abandoned during this period was higher the greater the distance to the nearest tree occupied by noctules (estimate = −79.93, SE = 33.13, χ^2^ = 5.82, P = 0.016) and the smaller the aggregation of trees occupied by noctules in 2013 (estimate = 0.23, SE = 0.11, χ^2^ = 4.07, P = 0.044). Interestingly, the probability of tree abandonment was also positively related to the presence of ring-necked parakeets nesting in the same tree in 2013 (estimate = 2.35, SE = 0.85, χ^2^ = 7.58, P = 0.006), and to the aggregation of trees occupied by parakeets around the tree previously occupied by noctules (estimate = −0.15, SE = 0.07, χ^2^ = 5.34, P = 0.021) (deviance explained = 24.30%). Indeed, 20 trees abandoned in 2013 by noctules were occupied by ring-necked parakeets, while only 2 of the trees that remained occupied by bats were also shared with the invasive species.

### Interspecific Aggressions

We recorded 435 encounters between nesting ring-necked parakeets and 13 bird species that approached within ≤15 m (Table 4). Three of them (blue-crowned parakeet, monk parakeet and Senegal parrot) were also exotic parrots. Four species were potential predators of adult birds or their eggs and nestlings (booted eagle, black kite, lesser kestrel and jackdaw), the last two also breeding in cavities (Table 4).

**Table 4:** Bird species that encountered nesting ring-necked parakeets during the 2013 breeding season in urban areas of Seville.

	Body mass	Predator	Cavity nester	Nesting overlap	# encounters
Ring-necked parakeet (*Psittacula krameri*)	116.5				
Spotless starling (*Sturnus unicolor*)	80	No	Yes	3	91
Feral pigeon (*Columba livia* var. *dom.*)	354,5	No	Yes	1	80
Lesser krestel (*Falco naumanni*)	152,5	Yes	Yes	2	73
House sparrow (*Passer domesticus*)	27,7	No	Yes	2	66
Jackdaw (*Corvus monedula*)	246	Yes	Yes	2	38
Senegal parrot (*Poicephalus senegalus*)	147	No	Yes	3	33
Eurasian collared dove (*Streptopelia decaocto*)	149	No	No	0	16
Blue-crowned parakeet (*Aratinga acuticaudata*)	165	No	Yes	3	10
Blue tit (*Cyanistes caeruleus*)	13.3	No	Yes	1	8
Black Kite (*Milvus migrans*)	827	Yes	No	0	7
Monk parakeet (*Myiopsitta monachus*)	101	No	No	0	7
Great tit (*Parus major*)	19	No	Yes	1	4
Booted eagle (*Aquila pennata*)	834.5	Yes	No	0	2

The average body mass of the species (in g), whether or not they can predate ring-necked parakeets (eggs, chick or adults) and are cavity nesters, as well as their overlap of nesting preferences and numbers of encounters are reported.

**Figure 2:**
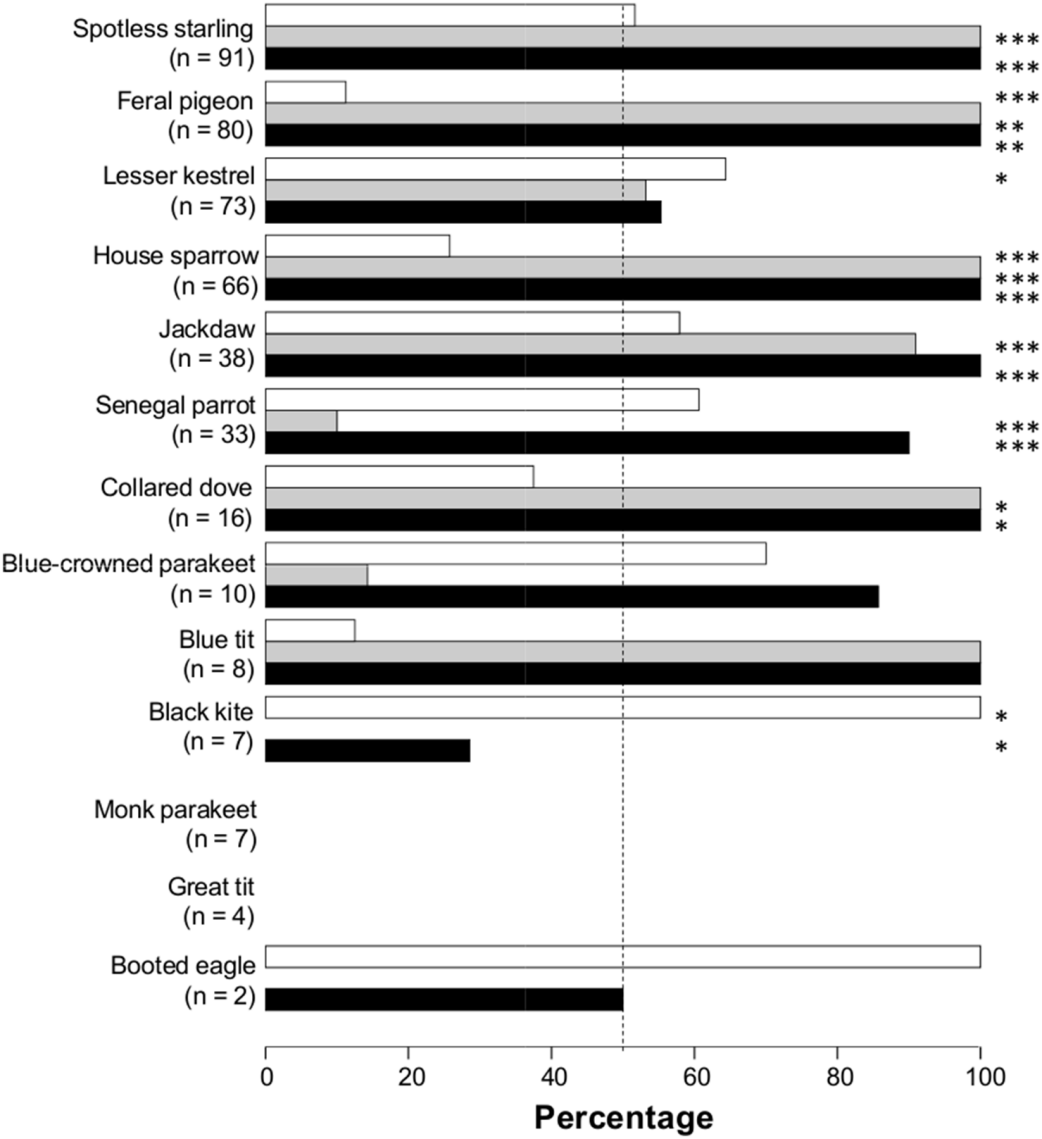
Percentage of encounters with ring-necked parakeets that ended in aggressions (white bars), and percentage of aggressions initiated (grey bars) and won by ring-necked parakeets (black bars). The number of recorded encounters is shown in brackets.

Almost half (42.5%, *n* = 435) of the encounters ended in aggressive interactions. Most aggressions were initiated by ring-necked parakeets (69.2%, *n* = 185; binomial test *P*<0.001), and this species won most of the fights (83.8%, n = 185, binomial test *P*<0.0001). However, the output of these encounters greatly varied among the interacting species (Figure 2). When considering the traits of the interacting species and the number of ring-necked parakeets and of the interacting species involved in encounters (Table 5), the probability that an encounter ended in aggression increased with the interspecific overlap in nest type preferences and the body mass of the interacting species, and decreased with the number of individuals of the interacting species involved. The probability that an aggression was initiated by ring-necked parakeets decreased with the body mass of the interacting species. Finally, the probability that a fight was won by ring-necked parakeets was greater if they initiated the attack but decreased when the interacting species was a potential avian predator. Nonetheless, ring-necked parakeets won 25–100% of the aggressions directed towards different predator species (Figure 2).

**Table 5:** GLMs explaining the probability that an interspecific encounter ended in aggression (Aggression), whether the aggression was initiated by ring-necked parakeets (Fight initiation) and was won by ring-necked parakeets (Win fight).

	Nesting overlap	N	Body mass	Attack initiation	Predator	% deviance
Aggression	78.83 (+)***	17.13 (−)***	27.24 (+)***			19.32
Fight initiation			21.89 (−)***			9.58
Win fight				24.70 (+)***	27.89 (−)***	37.02

The retained explanatory variables were the interspecific overlap in nest types (Nesting overlap), the number of individuals of the interacting species present in the encounters (N), the average body mass of the interacting species (Body mass), whether or not ring-necked parakeets initiated the aggression (Attack initiation), and whether or not the interacting species is a potential predator of birds. χ^2^ values are given for each variable. Signs between brackets indicate positive or negative effects of the explanatory variables. ***: p<0.001; % dev: percentage of deviance explained.

## Discussion

### Opportunism or Competition?

The successful establishment of exotic species in novel habitats constitutes a poorly understood paradox [35]. Recently, Sol et al. [15] examined the invasion paradox by studying the use of food resources by invasive and native bird species in an Australian city, concluding that the success of invaders is explained by their capacity to exploit ecological opportunities that most native species rarely use. However, as the authors pointed out, competition over other resources, notably nesting sites, must be considered in further studies [15].

We investigated two key aspects behind the establishment success of ring-necked parakeets on a relatively newly invaded urban area, namely: the way they shared nest-site resources with the recipient community and the aggressive interactions they experienced with other species. This approach allowed us to show that this species may invade new areas even when resources are not overabundant, thus not supporting the hypotheses proposing that saturated communities can halt biological invasions through competitive processes but rather supporting the competition hypothesis (instead of the opportunism hypothesis) for successful invasions [7]. Although the availability of tree cavities was relatively high in the study area compared to other cities [18], [36], the large populations sizes of different cavity-nester species together with the fact that the characteristics of unoccupied cavities differed from those of occupied ones suggest a shortage of suitable breeding sites for the native cavity-nesting community, coincident with the general pattern of competition found across cavity-nesting communities especially in urban environments [18], [36], [37]. Most of the inability of the native community to resist the parakeet invasion may be due to the invader’s highly aggressive behaviour that allows it to out-compete natives, thus successfully occupying areas even when there is no superabundant or underexploited resources. Interestingly, we were able to separate species into three main functional groups based on species-specific nest site requirements, showing that parakeets fit into one of these groups. Thus, even when they may be interacting with many native species, they share important resources for population prospects (i.e., reproduction) with only some of them. However, ring-necked parakeets were aggressive (and won most aggressive encounters) not only towards those species sharing nest-site preferences (including two other exotic parrot species) but also towards others, even non cavity-nesting species and avian predators. Therefore, the ring-necked parakeet has the potential to modify the numbers and spatial distribution of coexisting breeding species through behaviour-mediated competitive exclusion.

### Mechanisms behind the Spatial Arrangement of Species

Habitat selection models, and species distribution models in their broader sense, are mathematical descriptions of biological patterns that are affected by environmental conditions and a multitude of direct and indirect interactions [38], thus inferring that causal links from observational data should be made with caution. Two species may co-occur if they share their habitat requirements, but also if they facilitate each other directly or indirectly. Conversely, species may appear to avoid each other if they show competitive exclusion but also if they have dissimilar habitat requirements. Although competition for cavities can trigger intraspecific negative interactions among individuals, we detected a general tendency among species to breed following a pattern of conspecific aggregation. This seems not to result from heterogeneities in habitat and nest site availability, aspects which were controlled for in statistical analyses, and thus may rather be related to conspecific attraction processes as previously observed in many other colonial but also territorial species (e.g. [39], [40]). Different studies have shown that breeding in close proximity to conspecifics benefits breeders from earlier detection of predators, group defence, and dilution of predation [41]–[43].

Regarding the effects of the invasive species, we found that the spatial distribution of nesting ring-necked parakeets was important to explain the distribution patterns of all tree-cavity nester species of the recipient community while controlling for the main habitat features. However, the underlying putative mechanisms (attraction or segregation) were different among species. All bird species increased their likelihood of occupying cavities located close to parakeet nests and/or to high densities of parakeets. Positive co-occurrence patterns are indicative of heterospecific attraction [44], thus signaling the presence of direct or indirect species interactions. In our study system, a possible explanation for this association pattern could be found in the high aggressiveness of parakeets against avian predators. In fact, ring-necked parakeets may even communally attack predators, as we observed a flock of 60 parakeets mobbing a booted eagle (*Aquila pennata*) in María Luisa Park in 2008. Therefore, native species may choose breeding sites far enough from ring-necked parakeets (>15 m) to avoid aggressions but close enough to be rewarded by their effective anti-predator response, resulting in an active breeding association, which benefits the associated species [45]. Conversely, the mutual spatial segregation between parakeets and noctules, not explained by habitat features, could be indicative of direct competition since they share their preferences for the same kind of cavities. The nocturnal behaviour of bats precluded the systematic observation of encounters with ring-necked parakeets, which would have been restricted to instances when parakeets would enter bat cavities and inspect for potential nest sites. Although greater noctules aerially hunt small passerines when migrating at night [46], they are not able to kill birds inside their nests (J. Juste com. pers.) and even less so a much larger species such as the ring-necked parakeet whose body mass (116 g) is more than twice that of the noctule (50 g). Given that parakeets won most aggressions when encountering larger-bodied competitors such as feral pigeons and even powerful jackdaws (Figure 2), they would be expected to also win most aggressive interactions with this much smaller bat species. Although little is known about the effects of aggressive species like parakeets on mammals that shelter and reproduce in hollows like bats, several authors suggested that they can evict them [47], [48] and there is concern that ring-necked parakeets could cause the loss of suitable cavities for the noctule bat (*Nyctalus noctula*) in The Netherlands [49]. In our study area, a greater noctule was fortuitously observed being aggressively expulsed from its cavity by a ring-necked parakeet in María Luisa Park in 2005 (E. Revilla com. pers.), and it could be expected that the strong beak of parakeets could seriously injure noctules to the point of killing or impeding their flight by irreversibly damaging their sensible patagium (J. Juste com pers.). Moreover, there is a published observation of a similar body-sized exotic parakeet (Superb Parrot, *Polytelis swainsonii*) killing the much larger red squirrel (*Sciurus vulgaris,* 295 g [50]) in Italy [51] and evidence of similar cases that might had been caused by ring-necked parakeets in France [52], [53]. During this study we observed 11 instances of ring-necked parakeets (involving up to 10 individuals) attacking and mobbing black rats (*Rattus rattus* 180 g [50]), forcing them from the proximity of their nests. Since both rats and squirrels are predators of bird nests, including those of parakeets [51], these observations also reinforce the potential benefits to other bird species of breeding close to parakeets.

### Impact: Winners and Losers in a Contemporary Invasion Process

It is difficult to fully ascertain the ecological impacts of invaders, given the variety of potential impacts to be assessed, their subtle but pervasive effects, and the long time gaps between the introduction of an exotic species and its achievement of invasiveness and detectable impacts [54], [55]. Although there are well-recognized cases of negative impacts of bird invasions in island environments [56], [57], their impact on mainland environments have been less studied and much debated [58]–[60], to the point of suggesting that introduced bird species should be managed before their negative impacts are proven [61].

Although ring-necked parakeets have been shown to outcompete a small cavity-nesting native bird species in Brussels [18], there is little evidence of its impact on native communities when comparing areas occupied or unoccupied by this invader [22], [23]. Our different approach, by recording the output of inter-specific aggressions and the spatial distribution of species in a Mediterranean city, suggests, however, that ring-necked parakeets may trigger strong effects on the native recipient communities, with both positive and negative responses depending on the native species considered.

The spatial segregation of greater noctules and ring-necked parakeets together with the spatial patterns of trees abandoned during the last decade by noctules, its apparent population decrease and the parallel increase in the ring-necked parakeet population (Authors unpubl. data) suggest an active displacement exerted by the invasive species. This is a matter of concern for this bat species, which shows a scattered distribution throughout Europe and is classified as Vulnerable in Spain, with María Luisa Park supporting its largest known colony [62]. Previous radio-tracking studies showed that greater noctules forage over large extensions of natural habitats (up to 40 km from the urban park [63]), including Doñana National Park and surrounding marshlands, but they return daily for roosting to Maria Luisa Park and no alternative refuges are known for this population [27], [63]. This large population of greater noctules is therefore highly sensitive to any reduction in the availability of tree cavities caused by ring-necked parakeets. Given the scarcity of mature forests with large numbers of adequate cavities for the species, the other –although smaller- colony of greater noctules known in South Spain is also located in an urban park (in Jerez de la Frontera, 77 km far from Seville) [46]. Although the presence of ring-necked parakeets is still anecdotic in this city, its population expansion might also pose threats to this bat population in the near future. Further studies are needed to deep on the population ecology and trends of greater noctule populations, and of other bat species [49], related to their coexistence with invasive parakeets.

Another cause of concern is the fact that parakeets began to use wall cavities in 2011, breeding in three buildings in 2013, one of them located in the core of the city where there is also a colony of lesser kestrels. This colonial falcon suffered a drastic decline in Europe due to land-use changes that did not revert until recent years thanks to widespread conservation actions, including the provisioning of nest cavities [64]. Lesser kestrels breeding in Seville have to forage far from their breeding colony [65] but gain benefits by the reduced predation risk in the city [32]. Although their breeding success was linked to the quality of wall cavities [66], the species was not constrained by nest-site availability or competition with feral pigeons and jackdaws in recent decades [67]. However, the newly established ring-necked parakeet fought more than expected with lesser kestrels and won more than half of the aggressive encounters (Figure 2), while occupying only six wall cavities within the lesser kestrel colony. If the parakeet population continues to grow exponentially, it may pose a serious problem for urban lesser kestrels as well. In contrast to noctules and lesser kestrels, which are forced to forage far from the city, the abundance of food resources for ring-necked parakeets in the urban parks could reduce the energy they expend, allowing an increase in their breeding success and population growth [68], thus reinforcing their competitive superiority.

Both winner and loser species may result from anthropogenic-driven expansions of species [69], some invaders even favoring whole communities of natives [70]. Our results suggest that the presence of nesting ring-necked parakeets may benefit several non-threatened native bird species, which may incur breeding advantages by exploiting their effective anti-predatory behavior. However, this situation could change in the near future if the ring-necked parakeet population continues to grow. This is already the most abundant species breeding in the park and is the only one able to enlarge tree cavities up to reaching its preferred size (4–8 cm; [18,26, this study]), as has been shown in other urban parks [36]. In fact, 7 out of the 28 small-sized cavities (entrance <4 cm) were enlarged and occupied by parakeets during this study. Therefore, nest sites may become limited even for species using small-sized cavities such as tits and house sparrows. The latter species is a widespread commensal whose European populations are now decreasing, thus drawing attention to its long-term conservation status [71], [72].

### Conservation Implications

We have shown potentially serious impacts of an invasive bird targeting species that are not easily monitored or that are not expected to interact with them, such as a forest bat and a colonial falcon nesting in buildings, thus highlighting the difficulties in assessing the entire set of impacts posed by invaders [55]. The potential impact of ring-necked parakeets [18], as well as of other parakeet species [73] thriving in urban habitats, has been often discounted since urban bird communities are usually composed by few, generalist and non-threatened species [13], [15]. However, our case study suggests that urban ring-necked parakeets may be negatively affecting two threatened species, with some common species probably also affected in the near future if the parakeet population continues to grow. Therefore, both the conservation status of the native species with which the invader interacts as well as the population trends of the invader should be considered. Moreover, the positive population trends of ring-necked parakeets in Spain (authors' unpubl. data) suggests, as for monk parakeets [74], that the species could spread and invade rural habitats, as is already the case in central Spain (authors' unpubl. data). In such cases, parakeets would interact with a wider community of non-urban species and new impacts could arise, thus requiring a close monitoring of inter-specific interactions.

As recommended for other invasive organisms [55], management of avian invasions should be undertaken before populations spread and actions become costly and even unaffordable [61]. In this regard, our results provide evidence for the need of implementing control or even eradication plans for ring-necked parakeets in Spain. A very recent law (Real Decreto 630/2013) includes this species in the Spanish Catalogue of Invasive Species and provides legal coverage for such actions. We recognize that the success of these management actions is highly dependent on social perception, and projects involving eradicating birds are usually those least supported by citizens [75]. This is exacerbated in the case of the highly charismatic urban parrots [76]. Therefore, efforts should be made to raise public awareness of the problem [77], using for this purpose not only the ecological effects but also its potential economic and health impacts [78], [79].
